# Anthocyanins in Chronic Diseases: The Power of Purple

**DOI:** 10.3390/nu14102161

**Published:** 2022-05-23

**Authors:** Sunil K. Panchal, Oliver D. John, Michael L. Mathai, Lindsay Brown

**Affiliations:** 1 School of Science, Western Sydney University, Penrith, NSW 2753, Australia; s.panchal@westernsydney.edu.au; 2 Global Centre for Land-Based Innovation, Western Sydney University, Penrith, NSW 2753, Australia; 3 Faculty of Science and Natural Resources, Universiti Malaysia Sabah, Kota Kinabalu 88400, Sabah, Malaysia; oliverdjohn@outlook.com or; 4 Institute of Health and Sport, College of Health and Biomedicine, Victoria University, Melbourne, VIC 3021, Australia; michael.mathai@vu.edu.au; 5 Florey Institute of Neuroscience and Mental Health, Melbourne, VIC 3052, Australia; 6 School of Pharmacy and Medical Science, Griffith University, Gold Coast, QLD 4222, Australia

**Keywords:** anthocyanins, tropical fruit, agri-waste, inflammation, oxidative stress, gut microbiota

## Abstract

Anthocyanins are mainly purple-coloured phenolic compounds of plant origin that as secondary metabolites are important in plant survival. Understanding their health benefits in humans requires sourcing these unstable compounds in sufficient quantities at a reasonable cost, which has led to improved methods of extraction. Dark-coloured fruits, cereals and vegetables are current sources of these compounds. The range of potential sustainable sources is much larger and includes non-commercialised native plants from around the world and agri-waste containing anthocyanins. In the last 5 years, there have been significant advances in developing the therapeutic potential of anthocyanins in chronic human diseases. Anthocyanins exert their beneficial effects through improvements in gut microbiota, oxidative stress and inflammation, and modulation of neuropeptides such as insulin-like growth factor-1. Their health benefits in humans include reduced cognitive decline; protection of organs such as the liver, as well as the cardiovascular system, gastrointestinal tract and kidneys; improvements in bone health and obesity; and regulation of glucose and lipid metabolism. This review summarises some of the sources of anthocyanins and their mechanisms and benefits in the treatment of chronic human diseases.

## 1. Introduction

Anthocyanins are metabolites and distributed in flowers, fruits and vegetables [1]. They provide various colours such as red, pink, blue and purple. To date, more than 700 anthocyanins have been identified in nature [1]. These anthocyanins play a key role in plant reproduction by attracting pollinators and seed dispersers along with helping plants protect against many abiotic and biotic stresses [2,3]. Although these compounds have been studied for more than 100 years [4,5], their health benefits have only been widely recognised in the past 30 years. Firstly, this review will highlight methods of obtaining anthocyanins from standard and new plant sources, including agri-waste. The application of anthocyanins as food additives in the food chain is still restricted due to the difficulty in obtaining the molecules, which includes extraction, purification and stabilisation processes, despite functional and technological advances and marked economic potential [6]. Secondly, we will discuss the likely mechanisms of action of anthocyanins in the treatment of chronic disease in humans. We will emphasise human studies when available but some animal or cell studies will be included as they may initiate new understandings of mechanisms and improve concepts for interventions in chronic human diseases. Thirdly, while the health benefits of dietary anthocyanins in chronic diseases have been reviewed previously [7,8,9], we will show how recent studies have extended the therapeutic potential of interventions with anthocyanins in chronic human diseases. These recent studies are the basis for future investigations to improve human health using anthocyanins.

## 2. Sources of Anthocyanins

### 2.1. Structures and Recovery of Anthocyanins

**Figure 1:**
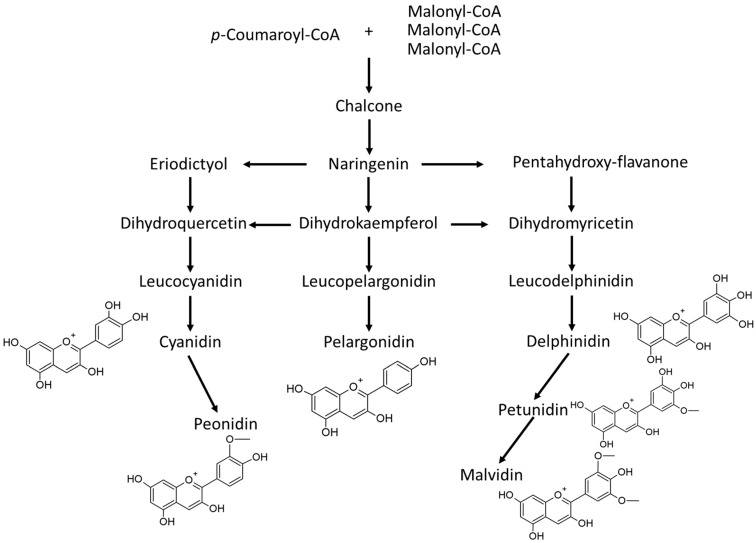
Biosynthesis of six important anthocyanidins [11].

Anthocyanins are composed of an anthocyanidin backbone (aglycone) with sugar and acyl conjugates. Pelargonidin, cyanidin and delphinidin are the major anthocyanidins while peonidin, petunidin and malvidin are the other important anthocyanidins found in nature [2]. The synthetic pathways to these anthocyanidins in plants are given in Figure 1. The sugars attached to anthocyanidins to form the anthocyanins are often glucose, arabinose, galactose and rutinose (6-O-α-L-rhamnosyl-D-glucose), often acylated [10].

Anthocyanin molecules are highly unstable and easily degraded by enzymes, light, temperature, pH and oxygen concentration; these factors prove a challenge to the recovery processes [6,12]. Method selection should consider the cost, versatility, method complexity, extractability and preservation of the target compounds [12,13]. An example of anthocyanin recovery from blackcurrant pomace is the use of acidified water extraction followed by a solid-phase extraction with sequential liquid–liquid aqueous-organic partitioning to separate monomeric and polymeric anthocyanins as well as flavonoids. The anthocyanins included delphinidin 3-rutinoside (45%) and cyanidin 3-rutinoside (31%), and the corresponding glucosides at 16% and 8%, respectively [14].

Increasingly, the food industry is interested in applying mild non-thermal methods of processing in anthocyanin extraction including ultrasound-assisted extraction, pressurised liquid extraction, supercritical fluid extraction, high hydrostatic pressure extraction, pulsed light, pulsed electric field, high-voltage energy discharge extraction, microwave-assisted extraction, enzyme-assisted extraction and instant controlled pressure drop-assisted extraction together with bio-based solvents [12,15,16,17,18].

The advantages of using ultrasound-assisted extraction processes are the maintenance of the biological activities of thermolabile bioactive compounds, lower cost and green and sustainable methods, mainly through reductions in solvent usage and time expenditure [19,20]. However, ultrasound processing can generate free radicals which promote degradation of bioactive compounds, for instance in red wine [21]. As an example, an ultrasound-assisted extraction method recovered ~23.4 mg anthocyanins/100 g of *Garcinia mangostana* peel, showing both that anthocyanin extraction is feasible and that the fruit waste product is a promising source of anthocyanins for functional foods or supplements [22].

Microwave-assisted extraction is another green extraction method that produces a high yield with decreased solvent usage and lower extraction time [23]. Microwave-assisted extraction improved extraction of anthocyanins from agri-food by-products compared to other traditional extraction technologies or other green extraction methods such as ultrasound-assisted methods [12]. However, an extraction temperature of more than 60–64 °C, irradiation times of more than 15 min and increased microwave power decreased yields of anthocyanins [24,25,26].

Other techniques include pressurised liquid extraction with organic solvents and/or water to extract functional compounds from fruits and vegetables, requiring less time under high temperature and pressure [27]. Pressurised liquid extraction has been extensively used to extract phytochemicals such as anthocyanins from berries [28].

Supercritical fluid extraction using carbon dioxide as a solvent has many advantages for phytochemical extraction, including its thermodynamic parameters and non-toxicity [29]. As an example, supercritical fluid extraction of black beans with carbon dioxide and water-ethanol as co-solvents increased the yield of polyphenols including anthocyanins for use as antioxidant and anti-ageing ingredients in cosmetics [30].

Enzyme-assisted extraction of some functional compounds from fruits has been widely used as it produces higher extraction efficiency than traditional extraction methods. Anthocyanin extraction from fresh grapes indicated that enzymes can soften cell walls, block the leakage of bioactive ingredients and increase the extraction rate of anthocyanins [31]. In addition, the combination of enzyme extraction and supercritical fluid extraction can improve the extraction of plant polyphenols while reducing degradation of the bioactive molecules [32].

Flash extraction is a physical method of extracting bioactive components from materials [33] that has not been widely applied to anthocyanin recovery.

Other extraction methods for anthocyanins include silica-based strong cation exchange solid-phase extraction combined with ultrahigh-performance liquid chromatography, ultrasound-microwave assisted extraction, deep-eutectic-solvent-based ultrasound-assisted extraction (deep eutectic solvents consist of two or more plant-based primary metabolites such as organic acids, sugars, alcohols, amines and amino acids), carbon dioxide expanded liquid extraction combined with sonication, dispersive solid phase extraction and high-hydrostatic-pressure-assisted extraction [28].

Treatment of proanthocyanidins in acidic and heating conditions is a further source of anthocyanins [34,35]. Proanthocyanidins are condensed tannins which are found in the berries, flowers, nuts, fruits, bark and seeds of various plants [34]. Proanthocyanidin monomers and oligomers up to a degree of polymerisation of four can be absorbed to produce antioxidant, cardioprotective, neuroprotective, lipid-lowering and antidiabetic effects [34].

### 2.2. Dietary Sources of Anthocyanins

The common dietary sources of anthocyanins are berries, cherries, peaches, grapes, pomegranates, plums, blackcurrants, red onions, red radishes, black beans, eggplants, purple corn, purple carrots, red cabbages and purple sweet potatoes [36]. The average daily intake of flavonoids in 1183 middle-aged Australian men and women was 625.51 ± 578.87 mg/day including 32.88 ± 24.22 mg/day anthocyanins with roughly half or 14.54 ± 12.07 mg/day as cyanidins [37]. The main sources of cyanidins were apples/pears (38%), canned fruits/fruits in juices/syrups (28%), berries (12%), plums (10%) and raisins/sultanas (9%). The bioavailability of flavonoids is quite low with only 1–2% of intact anthocyanins found after oral ingestion [38]. However, this value may be an under-estimate as bioactive metabolites from the gut microbiota may not be measured and anthocyanidin metabolites may be extensively degraded during storage of plasma before analysis [38]. Recent studies have compared the bioactivity of anthocyanins with their catabolites and gut microbiota metabolites. These studies have postulated that the bioactivity of anthocyanins results from exposure to a range of catabolites of anthocyanins, which can modulate inflammatory and cellular adhesion pathways [39,40].

Anthocyanin contents of these fruits and vegetables vary across plant species and cultivars along with differences introduced by environmental factors including light and temperature [36]. Berry fruits, the most popular and the richest source of anthocyanins, include blackberries (70.3 to 201 mg/100 g) [41], strawberries (20 to 60 mg/100 g) [42], blueberries (57 to 503 mg/100 g) [43], cherries (680 mg/100 g) [44], currants (0.205 to 2.253 mg/100 g) [45], chokeberries (141 to 2468 mg/100 g) [46], gooseberries (30 to 223 mg/100 g) [47], elderberries (670 mg/100 g) [44] and lingonberries (27.4 to 52.6 mg/100 g) [48]. The Saskatoon berry (*Amelanchier alnifolia*) native to Canada contains increased amounts of anthocyanins compared to many other berries (294 mg/100 g) [49]. The anthocyanins of these cold-climate berries are cyanidin 3-galactoside, cyanidin 3-glucoside and other cyanidin glycosides, delphinidin 3-glucoside, malvidin 3-glucoside and malvidin 3-galactoside [50,51]. Queen Garnet plums (*Prunus salicina* Lindl., a variety of Japanese plum) contain ~180 mg/100 g anthocyanins mostly as cyanidin 3-glucoside [52].

Apart from fruits and vegetables, anthocyanins are also present in black-, purple-, blue-, pink-, red- and brown-coloured varieties of cereals such as corn, rice, wheat, rye, barley and sorghum [53,54,55]. These coloured cereals can form a valuable supplement to standard human diets by providing both fibre and anthocyanins for additional health benefits.

### 2.3. Tropical and Subtropical Foods as Sources of Anthocyanins

Tropical fruit production and commercialisation have increased markedly, mainly in low-income areas such as Latin America and the Caribbean, with an estimated annual growth of 3.8% [56]. These fruits contain a wide variety of flavonoids, including anthocyanins. Anthocyanins are also found in many fruits from tropical and semi-tropical regions in South-East Asia and India; many tropical fruits are now grown commercially in countries such as Australia [57]. These fruits have found local use but they are underutilised as dietary components and potential therapeutic agents for humans. As an example, the characteristics of seven dark-coloured Brazilian fruits that are relatively unknown both to the population and industry have been described; in these fruits, anthocyanin concentrations range from 252 to 1610 mg cyanidin 3-glucoside equivalents/100 g dry weight [58].

One of the largest biodiversity areas in the world is the savanna in the Brazilian Cerrado. The jussara palm tree (*Euterpe edulis*) from this area is now considered an endangered species. Its fruit has high nutritional value and also contains an average of 369 mg anthocyanins/100 g with cyanidin 3-rutinoside (148 mg/100 g) and cyanidin 3-glucoside (56 mg/100 g) as the major anthocyanins [59]. Açai fruits (*Euterpe oleracea* Mart.) contain cyanidin 3-rutinoside (29–218 mg/100 g), cyanidin 3-glucoside (31–108 mg/100 g) and peonidin 3-glucoside (4–10 mg/100 g) [60]. The most common anthocyanins in Brazilian jaboticaba fruit are the glucosides of cyanidin (123–433 mg/100 g dry weight) and delphinidin (23.5–81 mg/100 g dry weight) as well as the galactosides of cyanidin (418.35 mg/100 g dry weight) and delphinidin (61.57 mg/100 g dry weight) [61].

Fruits from other tropical areas also contain anthocyanins. The anthocyanins in pomegranate (*Punica granatum* L.) include cyanidin 3-glucoside (22.77 mg/100 g), cyanidin 3,5-diglucoside (25.36 mg/100 g) and pelargonidin 3,5-diglucoside (11.16 mg/100 g) [62]. The anthocyanins are mainly concentrated in the aril and hence in the juice of the fruit [63]. Eggplant (*Solanum melongena* L.) peel contains anthocyanins, with the black bell cultivar containing the most anthocyanins at its ripe stage, including delphinidin 3-rutinoside (87.8 mg/100 g dried matter), delphinidin 3-rutinoside-5-glucoside (29.8 mg/100 g dried matter) and delphinidin 3-(p-coumaroyl)-rutinoside-5-glucoside (576.6 mg/100 g dried matter), with the overall total anthocyanins calculated as 694.1 mg/100 g dried matter [64]. Chinese bayberry cultivars (*Myrica rubra* Sieb. et Zucc.) contain several types of anthocyanins such as delphinidin 3-glucoside (0.87–1.11 mg/100 g), cyanidin 3-glucoside (0.93–91.22 mg/100 g), cyanidin 3-galactoside (0.88–3.54 mg/100 g), pelargonidin 3-glucoside (0.86–1.04 mg/100 g) and peonidin 3-glucoside (0.91–2.05 mg/100 g) in fresh weight [65]. The fruit has been used for its medicinal value and health-promoting qualities [66].

The dried pulp of Davidson’s plum (*Davidsonia pruriens*), a native fruit in Australia, contains anthocyanins (691 mg/100 g), particularly cyanidin glucoside, cyanidin sambubioside, peonidin sambubioside and peonidin glucoside [67]. Other Australian native fruits that contain anthocyanins include Tasmanian pepperberry (7920 mg/100 g), quandong (53 mg/100 g) and riberry (3543 mg/100 g) of anthocyanins equivalent to cyanidin 3-glucoside from the dry weight [68].

Traditional foods in tropical areas, such as Borneo, have the potential to be useful sources of anthocyanins. A popular vegetable salad from the fern family, the edible young sterile frond of *Stenochlaena palustris*, contains anthocyanins (51.3 mg/100 g of dry matter); it has been used traditionally as a herbal medicine for fever and postpartum recovery [69]. Another example is the butterfly pea flower (*Clitoria ternatea*), a popular ornamental and medicinal plant in Asia rich in anthocyanins (146 mg/100 g) [70], mainly polyacylated derivatives of delphinidin 3,3′,5′-triglucoside [71]. Wild berries from Borneo, another potential source of anthocyanins, include *Rubus moluccanus* (3696 mg/100 g), *Rubus fraxinifolius* (2227 mg/100 g) and *Rubus alpestris* (3362 mg/100 g) as cyanidin 3-glucoside equivalents [72,73].

In Thailand, local berries including *Prunus domestica* L. (4880 mg/100 g), *Antidesma bunius* L. Spreng (4950 mg/100 g), *Syzygium cumini* L. Skeels (4400 mg/100 g) and *Syzygium nervosum* A. Cunn. Ex DC (3790 mg/100 g) of extracts equivalent to cyanidin 3-glucoside are also promising sources of anthocyanins [74]. Local fruits, vegetables and flowers have been analysed for their anthocyanin constituents; among the notable ones are roselle (*Hibiscus sabdariffa* L., 120 mg/100 g of cyanidin 3-glucoside), common plum (*Prunus domestica* 1600 mg/100 g of cyanidin 3-glucoside), Ma Kiang (*Cleistocalyx nervosum var. paniala*, 2640 mg/100 g of cyanidin 3-glucoside), purple eggplant (*Solanum melongena* L., 2000 mg/100 g of delphinidin 3-glucoside), red grape (*Vitis vinifera* L., 1370 mg/100 g of peonidin 3-glucoside) and purple lettuce (*Lactuca sativa* L., 2180 mg/100 g of malvidin 3-glucoside), showing that food sources other than berries can be a feasible alternative for anthocyanins in the tropics [75]. Thai yam tubers contain 10–90 mg/100 g of anthocyanins cyanidin glucoside-equivalent; the anthocyanin types were found to be cyanidin-type anthocyanins and peonidin-type anthocyanins [76,77].

The Indian subcontinent contains several biodiversity hotspots with a wide range of edible fruits that have received little attention for their nutritional and health properties, for example the Western Ghats in southern India [78]. Mangosteens including *Garcinia indica* (kokum), native to this area, contain a wide variety of biologically active compounds including cyanidin 3-sambubioside and cyanidin 3-glucoside as well as garcinol [79]. In five fruits from the Indian Himalayan region, *Myrica esculenta*, *Berberis asiatica*, *Rubus ellipticus*, *Morus alba* and *Pyracantha crenulata,* the anthocyanin contents increased in the ripe stage of these fruits [80]. *Myrica esculenta* recorded the highest amount of anthocyanins with cyanidin (420 mg/100 g) and delphinidin (110 mg/100 g) [80]. The increased concentration of anthocyanins in ripened fruits could be attributed to the upregulation of the phenylpropanoid pathway and chalcone synthase involved in anthocyanin biosynthesis [81]. Anthocyanin profiling of blood fruit (*Haematocarpus validus)* naturally distributed in the Andaman and Nicobar Islands and the North-East States of India determined the concentration of anthocyanins in the pulp as 876 mg/100 g with pelargonidin as the dominant anthocyanidin [82]. *Syzygium cumini*, a tropical fruit originating in India, contained 210.9 mg/100 g total anthocyanins, which included delphinidin 3,5-diglucoside (95.6 mg/100 g), petunidin 3,5-diglucoside (68 mg/100 g), malvidin 3,5-diglucoside (32 mg/100 g), cyanidin 3,5-diglucoside (8.8 mg/100 g) and peonidin 3,5-diglucoside (4.7 mg/100 g) [83]. Extraction of these anthocyanins as a mixture from *Syzygium cumini* can be a valuable opportunity for the food, pharmaceutical and cosmetic industries [84,85]. Tuber vegetables are another potential source of anthocyanins such as sweet potatoes (*Ipomea batatas*) and purple yams (*Dioscorea alata*). Purple sweet potato has also been explored for anthocyanin composition and extraction (84–174 mg anthocyanins/100 g fresh weight). With the longer shelf-life of sweet potatoes, this root crop can provide a suitable source of anthocyanins in the diet [86,87]. Using these wild fruits as a source of anthocyanins, knowing their optimum harvest time, would allow more extensive dietary usage as well as nutraceutical and functional food applications [80].

### 2.4. Agri-Waste as a Source of Anthocyanins

Phenolic compounds are retained in the food matrix during processing and usually discarded as waste, which could have high valorisation potential. The economic losses and environmental problems caused by waste can be reduced by applying efficient agri-food by-product recovery. Waste products from fruits usually include the peel, leftover pulp, seed and stems not consumed by humans [88,89]. This is an important resource as food by-products such as peels, stems, trimmings, bran, shells and seeds often account for more than half the weight of fresh fruit and usually contain higher nutritional and functional content than the edible part [90].

Grapes are one of the richest sources of polyphenols including flavonoids, anthocyanins and proanthocyanidins. Grape by-products especially from wine production are useful sources of anthocyanins (25.89 mg/100 g), with the major anthocyanins being malvidin 3-glucoside (15.2 mg/100 g), delphinidin 3-glucoside (4.88 mg/100 g) and petunidin 3-glucoside (3.83 mg/100 g) [91]. Wine pomace, with anthocyanins and fibre content, can provide a valuable raw material for product development that can provide health benefits [92]. Among the Vaccinium berries (bilberries, blueberries, lingonberries and cranberries), the freeze-dried press residues from bilberries contained the highest amount of anthocyanins (28,495 mg/100 g) followed by blueberries (8412 mg/100 g), cranberries (4353 mg/100 g), and lingonberries (2758 mg/100 g) [93]. The anthocyanins from these residues were glycoconjugates of delphinidin, cyanidin, petunidin and malvidin [93]. Elderberry, *Sambucus nigra* L., branch waste contained anthocyanins (235 mg/100 g) as cyanidin and its glycoconjugates [94].

Passion fruit peel contains 577.6 mg/100 g anthocyanin as cyanidin 3-glucoside from the dry weight [95]. Total anthocyanin in red banana peel is 15.5 mg/100 g, which is higher than yellow banana peel (2.37 mg/100 g) [96]. In addition, banana bracts contain anthocyanins 32.3 mg/100 g as cyanidin 3-rutinoside equivalent; the anthocyanins identified include cyanidin 3-rutinoside and other 3-rutinoside derivatives of pelargonidin, delphinidin, peonidin and malvidin, which have been extracted using various solvent methods [97,98]. In addition to a high amount of ascorbic acid, acerola (*Malphigia emarginata* DC.), also known as Barbados cherry or West Indian cherry, contains anthocyanins [99]. However, the industrial residues of acerola (seeds) usually discarded as waste contain higher concentrations of anthocyanins (245.90 mg/100 g) than the pulp (144.27 mg/100 g) [100]. Another potential source of anthocyanins from tropical fruits is the rind of purple mangosteen, *Garcinia mangostana*, with an anthocyanin amount of about 137 mg/100 g from dried peel [101]. Apart from containing dietary fibre and phytochemicals, apple pomace, which is a by-product of apple juice processing, contains monomeric anthocyanins [102] and its content has been reported to be between 5–13 mg/100 g as cyanidin 3-galactoside [103].

The peel of the fruit of the Brazilian tree, the jaboticaba (*Myrciaria jaboticaba*), contains anthocyanins (107 ± 3 mg/100 g), mainly cyanidin glucoside (61 ± 0.51 mg/100 g) and delphinidin glucoside (8 ± 0.21 mg/100 g) [104]. Other less well-known fruits as sources of anthocyanins include black sapote (*Diospyros digyna* Jacq.), durian (*Durio zibethinus* L.), jaboticaba (*Plinia caulifora* Mart.) and Mora de Castilla (*Rubus glaucus* Benth.) [103].

These fruits, vegetables and waste products, mainly from tropical areas, provide local and sustainable options to increase the dietary consumption of anthocyanins [105]. The assumption that this increased intake will mitigate chronic diseases will be considered in the next section.

## 3. Mechanisms of Action of Anthocyanins in Disease

**Figure 2:**
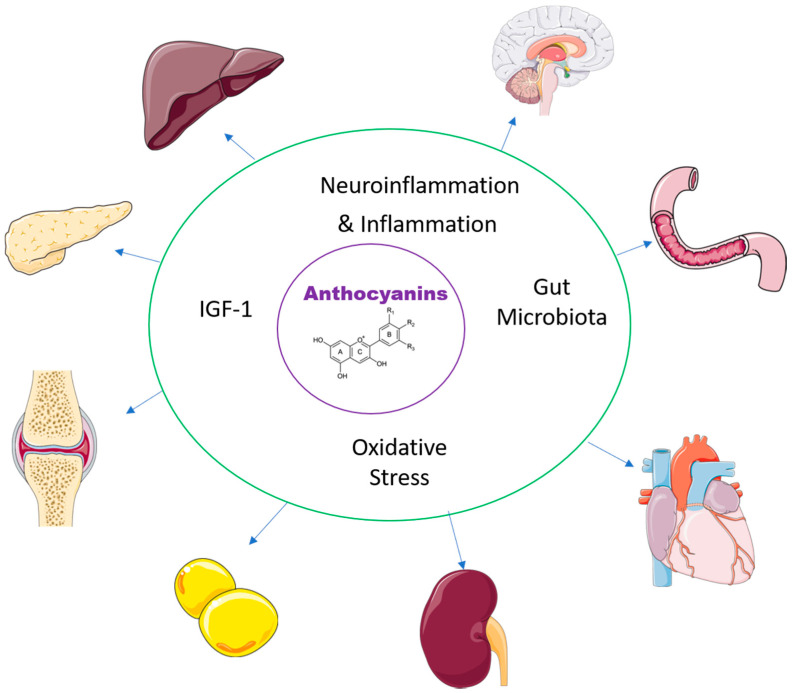
Effects of anthocyanins in the major organs.

Functional foods containing anthocyanins have been studied for health benefits but most of these studies have been in cells or animal models of human disease, rather than in human clinical trials. In this section, the recent literature has been reviewed to summarise the potential mechanisms of anthocyanins in chronic diseases such as changes in the gut microbiota, decreased oxidative stress, decreased inflammation and increasing insulin-like growth factor 1 (IGF-1) (Figure 2). The multiple changes in chronic disease states and the interactions between these mechanisms indicate that it is unlikely that any one mechanism is responsible for therapeutic responses in any particular disease state. However, an improved understanding of these mechanisms may allow more logical therapeutic choices to be made.

### 3.1. Gut Microbiota

After ingestion, functional foods first interact with the microorganisms in the gastrointestinal tract, collectively known as the gut microbiota. This complex micro-ecosystem is essential for many functions including digestion, regulation of the immune system and stabilising the intestinal barrier. Nutrients change the microbiota and intestinal barrier function, for example by the synthesis of folate to increase methylation and so change gastrointestinal function by epigenetic modifications [106]. Further, the gut microbiota may metabolise the functional foods. These microbial metabolites may produce physiological responses in the intestine and after absorption and distribution in the body. However, the metabolites may also lead to the development of chronic low-grade inflammation both in the intestine and throughout the body [107]. The bacterial metabolites that influence the immune signalling pathways include short-chain fatty acids, indole and indole acid derivatives, choline, bile acids, N-acyl amides, vitamins and polyamines [108]. Under physiological conditions when the inflammatory processes have achieved their aims, the resolution of inflammation is mediated by pro-resolving lipid mediators produced in damaged tissues, giving a further potential intervention to reduce inflammation through activating these pro-resolving pathways [109]. Changes in the gut microbiota have been suggested as being responsible for a wide range of diseases such as gastrointestinal diseases including inflammatory bowel disease [110], neurological disorders [111] including schizophrenia [112] and Parkinson’s disease [113], metabolic syndrome [114], cardiovascular disease [115], chronic kidney disease [116] and type 2 diabetes (termed diabetes in this review) [117], providing a potential target for the management of these diseases.

Anthocyanins can be absorbed in the stomach by specific transporters such as sodium-dependent glucose co-transporter 1, metabolised by the gut microbiota in the colon and modified by phase I and II metabolic enzymes in liver cells followed by enterohepatic circulation before distribution of anthocyanins and derivatives and accumulation in tissues [118]. These metabolites could independently alter organ structure and function in chronic disease states; for example, a major metabolite of anthocyanins, protocatechuic acid, has demonstrated antioxidant, anti-inflammatory and neuroprotective properties [119]. Concentrations in studies with isolated cells were usually around 10–100 μM while daily doses in rodents were around 10–100 mg/kg. Pharmacokinetic studies in humans after an oral dose of 500 mg cyanidin 3-glucoside show that serum concentrations of protocatechuic acid and its metabolites peaked at around 0.1 μM but were present in the circulation for longer and at higher concentrations than the parent anthocyanin [120]. In addition, protocatechuic acid may decrease cognitive and behavioural impairment, neuroinflammation and excessive production of reactive oxygen species [121].

Anthocyanins also act as prebiotics to modify the microbiota, in particular enhancing *Lactobacillus* spp. and *Bifidobacterium* spp. [122]. Both effects potentially lead to cardioprotective and neuroprotective responses and decreased bone loss in the ageing population [123]. Regulation of the gut microbiota by polyphenols, including anthocyanins, may reduce kidney injury to treat pre-existing chronic kidney disease [124]. Anthocyanins such as cyanidin 3-glucoside may be potential prebiotics, as purified cyanidin 3-glucoside (7.2 mg/kg/day) and anthocyanin-containing Saskatoon berry (8.0 mg/kg/day) administration for 11 weeks reduced high-fat high-sucrose diet-induced changes in the gut microbiota in mice [125].

### 3.2. Oxidative Stress

Low concentrations of reactive oxygen and nitrogen species (ROS/RNS) such as hydrogen peroxide, superoxide anions, hydroxyl radicals, nitric oxide and peroxynitrate are important in the defence against pathogenic microorganisms. Higher concentrations cause cellular damage leading to cell death by actions on DNA, proteins and lipids, a condition known as oxidative/nitrative stress. Oxidative stress is a key activator of disease onset and progression, overlapping with inflammation, providing the rationale for the effectiveness of anthocyanins in obesity, cardiovascular and neurological diseases [126].

Mitochondria are the major source of intracellular ROS as leakage of electrons through the respiratory chain. Accumulation of ROS in mitochondria disrupts normal function leading to depolarisation of the mitochondrial membrane. In high-energy-consuming cells such as cardiomyocytes, impaired mitochondrial activity will interfere with glucose and fatty acid metabolism leading to cardiomyopathy. Anthocyanins and their metabolite, protocatechuic acid, could decrease mitochondrial ROS concentrations and so reduce damage [127]. Further, some anthocyanins including cyanidin 3-glucoside may form an additional transport chain in damaged mitochondria as electron acceptors at Complex I to reduce cytochrome C and increase ATP production [128].

An additional target to reduce oxidative stress is DNA methylation as it is important in the long-term regulation of gene expression. Plant-derived antioxidants such as anthocyanins may be involved in epigenetic mechanisms to reverse aberrant methylation and oxidative stress without changing the underlying gene sequences [129]. These diseases occur through the regulation of epigenetic enzymes and chromatin remodelling complexes. Potential therapeutic targets include diseases that are increased in patients with metabolic syndrome, including atherosclerosis, diabetes, cancer and Alzheimer’s disease [129].

Further, mitochondrial DNA may be displaced from cells by cell-death-triggering stressors into extracellular compartments. This defective mitochondrial quality control may increase low-grade chronic inflammation by NLRP3 and other pathways [130]. This could provide pathways for compounds such as anthocyanins to improve mitochondrial function and decrease inflammation.

### 3.3. Inflammation

Low-grade chronic inflammation underlies many chronic systemic diseases, especially age-related decline and metabolic disorders [131,132,133,134,135,136]. In obesity, it is characterised by the secretion of a complex range of pro- and anti-inflammatory cytokines from expanding adipocytes in visceral adipose tissue known as adipokines that initiate and sustain the inflammation [137]. These adipokines impact remote organ function to produce the complications of cardiometabolic disease [138].

The bacteria in the gut microbiota regulate the permeability of the intestines with some species promoting a “leaky gut”. This allows microbial metabolites and components of the bacteria, such as lipopolysaccharides, to enter the circulation and start an inflammatory reaction by the release of cytokines. Further, gut microbiota allow the conversion of complex carbohydrates, not digested by the host, to short-chain fatty acids such as butyrate which are absorbed and may be anti-inflammatory [139]. As increased inflammation is associated with many diseases such as cardiovascular disease, diabetes, obesity, inflammatory bowel disease and cancer, understanding the role of the gut microbiota in these diseases is important.

In obesity, increased inflammatory responses occur with pro-inflammatory macrophages accumulating in adipose tissue, possibly with hypoxia as the initiating event leading to greater expression of hypoxia-inducible factor 1-α and increased inflammatory responses in the liver, pancreatic islets and gastrointestinal tract [140]. Adipose tissue inflammation decreases remote organ function, considered causative for the complications of obesity [138]. The role of the hypothalamus in the regulation of energy homeostasis is well-known. The inflammatory activation of glial cells in the hypothalamus leads to changes in feeding habits, thermogenesis and adipokine signalling, leading to metabolic disorders [141]. Further, maternal obesity and inflammation may lead to metabolic reprogramming in the foetus, which could influence both childhood and adult body weight and composition, thus increasing the risk of transgenerational transmission of obesity [142].

The anti-inflammatory responses to anthocyanins have been shown in many in vivo and in vitro systems; further, anthocyanins regulate pro-inflammatory markers in both healthy and chronic disease states [143]. There are many mechanisms for the anti-inflammatory effects of anthocyanins that could be applicable in chronic inflammatory diseases, including inhibiting the release of pro-inflammatory factors, reducing TLR4 expression, inhibition of the NF-κB and MAPK signalling pathways, and reducing the production of NO, ROS and prostaglandin E_2_ [144].

Reducing inflammation in the brain may play a role in anthocyanin-induced changes in chronic disease. Prolonged neuroinflammation damages brain function, possibly causing and accelerating long-term neurodegenerative diseases including dementia [145]. The gut-microbiota-brain axis allowing bidirectional communication is important in maintaining the homeostasis of the central nervous system as well as the gastrointestinal tract. Thus, much recent research has focused on the relationships between gut microbiota and neurological disorders such as schizophrenia and autism spectrum disorder and neurodegenerative disorders such as Alzheimer’s disease, Parkinson’s disease and ischaemic stroke, which involve the death of vulnerable populations of neurons [146]. Dysbiosis of the gut microbiota has been associated with the progression of both systemic inflammation and neuroinflammation [147]. Mediators of neuroinflammation include microglia, astrocytes and oligodendrocytes as well as damaged and dysfunctional mitochondria [148].

Anthocyanins and their metabolites produce neuroprotective activities by decreasing neuroinflammation, preventing excitotoxicity, preventing aggregation of proteins, activating pro-survival pathways while inhibiting pro-apoptotic pathways and improving axonal health [149,150]. The wide range of potential mechanisms to overcome neurotoxicity with anthocyanins such as cyanidin glucoside includes suppression of c-Jun N-terminal kinase activation, amelioration of cellular degeneration, activation of the brain-derived neurotrophic factor signalling and restoration of Ca^2+^ and Zn^2+^ homeostasis [151]. Blueberry extract (150 mg/kg/day) containing anthocyanins promoted neuronal autophagy to decrease neuronal damage in transgenic APP/PS1 mice with mutations associated with early-onset Alzheimer’s disease. Further, protocatechuic acid may be the major metabolite producing neuroprotection [152]. Possible changes in amino acid metabolism have been suggested as the mechanism of improved attention, feelings of alertness and mental fatigue after 3 months’ treatment with tart cherries containing anthocyanins and other polyphenols in middle-aged adults [153]. These changes could be beneficial if they can be translated to patients with neurological or neurodegenerative diseases.

However, most studies have been performed in cell culture or in pre-clinical animal models. Thus, the roles of anthocyanins and their metabolites in existing neurodegenerative diseases in humans where significant neuronal loss has already occurred are uncertain but possible. Targeting neuroinflammation with anthocyanins is therefore a promising strategy in the treatment of neurological disease [150] although the translation of results from animal models to patients with neurological disease requires further research. Further, direct pharmacological actions in the brain of orally administered anthocyanins require that anthocyanins pass through the blood–brain barrier, yet the permeability of anthocyanins has been shown to be low [154]. This suggests that the major reason for decreased neurotoxicity with anthocyanins could be that changes in gut microbiota lead to decreased neuroinflammation.

### 3.4. IGF-1

The neuropeptide IGF-1 has important roles in the development and maturation of the brain [155]. Cyclic glycine-proline (cGP), a neuropeptide formed from the N-terminal tripeptide fragment of IGF-1, activates and normalises IGF-1 essential for body growth, neurological function and lifespan [156,157]. However, IGF-1 may play opposing roles in the ageing brain, where chronic neurodegenerative, cardiovascular and metabolic diseases are more likely to have a pathological effect [158]. The study of the interactions of IGF-1 and anthocyanins is relatively recent but it is providing a potential mechanism for their therapeutic benefits.

The interaction of cGP with bound/free IGF-1 may mediate the therapeutic benefits of anthocyanins in reducing blood pressure and improving cognitive health [159]. cGP has a higher binding affinity to the IGF binding protein-3 (IGFBP-3) than IGF-1 itself, thereby increasing the free concentrations of active IGF-1 in the plasma. IGF-1 reduced hypertension in pre-clinical models of hypertension [160]. This could account for the normalisation of blood pressure in obese adults with mild hypertension [161].

Decreased peripheral and cerebrospinal fluid IGF-1 concentrations could be a potential marker for the cognitive decline and progression of Alzheimer’s disease. In the brain of Alzheimer’s disease patients, bioavailable IGF-1 deficiency was shown with increases in bound IGFBP-3 and bound cGP [162]. As IGF-1 is involved in synaptogenesis, increased bioavailable IGF-1/cGP could improve brain function in Alzheimer’s disease. Consumption of blackcurrant supplement containing 35% anthocyanins at a dose of ~600 mg daily increased cGP concentrations in both the plasma and cerebrospinal fluid in humans, showing that the lipophilic nature of cGP enabled rapid transfer across the blood–brain barrier [163]. This could support the hypothesis that anthocyanins are effective both in reducing blood pressure and improving brain function by increasing IGF-1 and cGP in the brain. The mechanisms through which dietary anthocyanins increase cGP in plasma and cerebrospinal fluid are currently under investigation. One study showed that blackcurrant juice contained both anthocyanins and cGP [163].

## 4. Therapeutic Actions of Anthocyanins in Chronic Diseases

### 4.1. Delaying Cognitive Decline

Cognitive decline is a characteristic of ageing including three major features of immunosenescence as inflamm-ageing, vascular ageing and brain ageing [164]. As inflammation is the common underlying mechanism, anti-inflammatory flavonoids may have a role in preventing cognitive decline. Higher flavonoid intakes were associated with a decreased subjective cognitive decline in US men and women with the strongest associations for flavones, flavanones and anthocyanins [165]. Supplementation with fish oil, blueberries or a combination for 24 weeks was given to 76 patients aged 62–80 years with self-perceived cognitive decline. Both individual interventions produced fewer cognitive symptoms and the combination improved memory discrimination [166]. Further, older adults with mild cognitive impairment at risk of dementia improved working memory after a 16 week intervention with blueberries with a daily dose of 269 mg cyanidin 3-glucoside equivalents [167]. In 49 patients over 70 years old with mild to moderate dementia, 12 week intervention with cherry juice containing anthocyanins improved verbal fluency, short-term memory and long-term memory, together with lowered systolic blood pressure [168]. These results suggest a role for the anti-neuroinflammatory responses of anthocyanins in moderating cognitive decline in older adults. Human intervention studies suggest that daily strawberry or blueberry intakes may improve cardiovascular and metabolic parameters and possibly improve cognitive function [169]. Further, modifications in the gut microbiota were partially linked to the neuroprotective properties of a blackberry anthocyanin-rich extract in Wistar rats fed a high-fat diet [170].

### 4.2. Modulating Neurodegenerative and Neurological Diseases

Neurodegenerative disorders such as Parkinson’s disease, Alzheimer’s disease and amyotrophic lateral sclerosis are characterised by the death of neurons and loss of signalling networks leading to impairment of both cognitive and motor function. Neuroinflammation is an important aspect of neurological and neurodegenerative diseases and the role of anthocyanins to reduce neuroinflammation has been outlined in Section 3.3. Further, the effects on gut microbiota, oxidative stress and inflammation suggest that anthocyanins could be neuroprotective, as shown in animal models of neurological and neurodegenerative diseases, and in studies using cell cultures [151].

Parkinson’s disease is a slowly developing neurodegenerative disorder with accumulating disability and no current therapy to slow down disease progression [171]. Observational studies have suggested that dietary antioxidants in humans may decrease the risk of Parkinson’s disease but only two of these studies involved anthocyanins to give a pooled relative risk of 0.76 [172]. Potential mechanisms include reduction of antioxidant stress, reducing excitotoxic insults, reducing neuroinflammation, preventing protein aggregation and endoplasmic reticulum stress and reducing apoptosis [149]. However, these studies have mostly been performed in animal models or cell cultures rather than in patients with Parkinson’s disease. The development of Parkinson’s disease has been associated with IGF-1, especially increased concentrations at the onset of the disease [173]. Cerebrospinal fluid concentrations of IGF-1 and cGP in Parkinsonian patients were increased after supplementation with blackcurrant anthocyanins, suggesting that anthocyanins may modulate IGF-1 function and thereby improve disease state [163].

The role of neuroinflammation, especially microglial activation, in the trajectory of Alzheimer’s disease suggests possible therapeutic options for anthocyanins [174]. Disturbances of the gut microbiota may worsen signs of Alzheimer’s disease by activating signalling pathways such as the TLR4/NF-κB, ROS/JNK and NF-κB/BACE1 pathways; inhibiting these pathways with anthocyanins was suggested to decrease neuroinflammation and Alzheimer’s pathology [175]. In a mouse model of Alzheimer’s disease, altered gut microbiota were associated with an increased NLRP3 inflammasome, enhanced astrogliosis and microglial activation [176]. Transplantation of the gut microbiota from Alzheimer’s disease patients into APP/PS1 double transgenic mice as a model of the disease increased expression of NLRP3 and led to more severe cognitive impairment [177]. Further, in mouse models of Alzheimer’s disease, remodelling of the gut microbiome with sodium oligomannate suppressed neuroinflammation to decrease disease progression [178]. A clinical trial in 818 mild-to-moderate Alzheimer’s disease patients with sodium oligomannate for 36 weeks showed sustained improved in cognition [179], demonstrating the potential of this intervention. As anthocyanins may reduce neuroinflammation [149,150,151], they could be useful options for Alzheimer’s disease by remodelling the gut microbiome. However, improvements in patients with Alzheimer’s disease given anthocyanins have not been reported. In contrast, dietary consumption of strawberries has been associated with a decreased risk of Alzheimer’s dementia [180].

Amyotrophic lateral sclerosis is characterised by neuron apoptosis followed by skeletal muscle atrophy [181] with proposed mechanisms including oxidative stress, neuroinflammation and mitochondrial dysfunction. While no reports on the treatment of human amyotrophic lateral sclerosis with anthocyanins have been found, the anthocyanin metabolite, protocatechuic acid, sustained neuromuscular function with improved motor function and decreased gliosis in the hSOD1^G93A^ mouse model of this disease [182].

Few studies have investigated the roles of anthocyanins to treat neurological disease in humans. Human studies using anthocyanins in insomnia, anxiety or depression suggest potential therapeutic benefits but reported studies are few and the patient numbers were low [183]. In eight patients aged over 50 years, tart cherry juice improved both sleep duration and efficiency with procyanidin B2 proposed as the major bioactive compound [184]. Further, patients with Parkinson’s disease scored lower on anxiety and depression scores after administration of blackcurrant concentrate (300 mg twice daily for 4 weeks) increased cGP concentrations in cerebrospinal fluid [163]. Clearly, further studies in humans with anthocyanins or their metabolites in these prevalent neurodegenerative and neurological diseases are necessary.

### 4.3. Protecting the Liver

The liver is an important organ for lipid metabolism as it plays vital roles in fatty acid synthesis and lipid circulation through lipoprotein metabolism [185]. Anthocyanins have shown beneficial effects on these lipid metabolic pathways in the liver. Mulberry anthocyanin extract in human hepatoma cells HepG2 reduced fatty acid synthesis and increased fatty acid oxidation in response to lipid accumulation by oleic acid. Anthocyanins from mulberry extract also inhibited acetyl coenzyme A carboxylase through AMP-activated protein kinase [186]. These responses were associated with reduced expression of sterol regulatory element-binding protein-1 (SREBP-1), fatty acid synthase, glycerol 3-phosphate acyltransferase, 3-hydroxy-3-methyl-glutaryl CoA reductase, adipocyte-specific fatty acid binding protein and SREBP-2 along with increased expression of peroxisome proliferator activated receptor α and carnitine palmitoyl-transferase-1 [186]. Similar results were obtained with delphinidin 3-sambubioside (100 or 200 μg/mL) from *Hibiscus sabdariffa* L. in HepG2 cells induced with oleic acid [187].

Non-alcoholic fatty liver disease (NAFLD) is a multifactorial disease defined as the accumulation of triglycerides in hepatocytes in the absence of ethanol consumption. It has a global prevalence of 25% yet no approved drug therapy [188]. Anthocyanins may decrease NAFLD by changing the gut microbiota, providing antioxidant and anti-inflammatory responses and improving lipid and glucose metabolism to reduce risk factors [189]. As an example, delphinidin 3-sambubioside (15 or 30 mg/day) in high-fat-diet-fed rats reduced body weight gain, visceral fat, abdominal fat and hepatic lipid deposition [187]. In patients with NAFLD, intervention for 12 weeks with a bilberry and blackcurrant mixture containing 320 mg anthocyanins improved insulin resistance, indicators of liver injury and clinical evolution [190]. However, meta-analysis of 12 randomised clinical trials in patients with metabolic disorders did not show significant effects of anthocyanin supplementation on the liver enzymes, ALT and AST [191].

### 4.4. Protecting the Heart and Blood Vessels

Hypertension remains the leading cause of cardiovascular disease and premature death, with an increased incidence in low- and middle-income countries [192]. As such, reducing cardiovascular disease using nutraceuticals has an obvious appeal. Anthocyanins reduced the risk of coronary heart disease and cardiovascular disease mortality but had no effect on myocardial infarctions, stroke or total cardiovascular disease [193]. A further analysis confirmed the decreased risk of cardiovascular disease with evidence that the protection could involve improved lipid profiles and decreased circulating pro-inflammatory cytokines [194]. An umbrella review showed a reduced risk of hypertension and improved markers of cardiometabolic disease but no effect on systolic or diastolic blood pressures [195]. However, a small observational study reported decreased blood pressures in mildly hypertensive patients given Queen Garnet plum juice containing 255 mg cyanidin 3-glucoside equivalents for 12 weeks [161].

The major sites of cardiovascular action of anthocyanins appear to be the vasculature rather than myocardial tissue. In rats with left anterior descending artery occlusion and reperfusion, cyanidin 3-glucoside at doses of 10 and 20 mg/kg/day protected heart tissue from ischaemia-reperfusion injury by attenuating oxidative stress and ferroptosis-related protein expression [196].

Atherosclerosis is an inflammatory vascular disease [197]. Oxidative stress and inflammatory signalling in cells in the atherosclerotic plaque such as macrophages and endothelial cells can be reduced by direct antioxidant actions, by inducing intracellular Nrf2 activation and antioxidant gene expression and by anti-inflammatory responses such as increased antioxidant capacity of HDL and decreasing lipid/protein oxidation [198]. Physiologically relevant concentrations of cyanidin 3-glucoside and its metabolites decreased expression of the inflammatory mediators, IL6 and VCAM-1, in human vascular endothelial cells in culture [199]. In hydrogen peroxide and LPS-stimulated diabetic human aortic endothelial cells, berry anthocyanins at 50 μL/mL reduced oxidative stress and inflammation by inhibition of the NF-κB signalling pathway [200].

A preliminary event in the development of atherosclerosis is the increased apoptosis of endothelial cells [201]. Cyanidin 3-glucoside decreased inflammation, suppressed apoptosis, lowered blood lipid concentrations and improved artery wall structure and function in rabbits fed a high-fat diet [202]. Berry anthocyanins may protect the vasculature in cardiometabolic disease by inducing NO production and decreasing inflammation and oxidative stress [203] as well as changes in the gut microbiota [204]. Further, Queen Garnet plum juice containing approximately 250 mg cyanidin 3-glucoside reduced the postprandial effects of a high-fat high-energy meal on vascular endothelial function and inflammatory responses in older humans [205]. Platelet aggregation is more likely following endothelial damage in humans. Platelet aggregation in vitro was decreased in healthy subjects following 21 day supplementation with Queen Garnet plum juice containing approximately 200 mg cyanidin 3-glucoside [206].

### 4.5. Maintaining Glucose Homeostasis

Type 2 diabetes is a chronic disease defined by hyperglycaemia leading to microvascular and macrovascular damage. The prevalence of diabetes has been increasing globally since 1990, attributed to living environments and lifestyle leading to poorer nutrition and increased sedentary behaviour [207]. Diabetes is more prevalent in people aged 65 years and older including 122 million of this population of 652 million, or around 19%; further, prediabetes affects 48% of the 26 million older adults in the USA. The risk to these patients is increased by multiple comorbidities, increased incidence of hypoglycaemia, increased dependence on care and worsening frailty [208]. A marked decrease in all-cause mortality rates of 30–35% in diabetics in the USA and England has been related to decreases in mortality from cardiovascular disease. Cancer rates have remained unchanged in diabetes patients but this is now an increased percentage of deaths while mortality rates from dementia and liver disease have increased [209]. Treatment of diabetes now includes glucagon-like peptide-1 receptor agonists and sodium-glucose cotransporter-2 inhibitors, with potential clinical use of adiponectin and fibroblast growth factor 21, so that these compounds will allow personalised approaches to lower blood glucose concentrations [210].

Large prospective cohort trials in the USA have shown an inverse relationship between a healthy plant-based diet and the risk of developing diabetes [211]. Further, diets containing polyphenols such as anthocyanins may reduce the risk of developing diabetes [212]. In clinical trials in diabetes, anthocyanins reduced blood glucose and HbA1c concentrations and improved insulin secretion and resistance [212,213]. Various studies have identified beneficial impacts of dietary polyphenolic compounds including anthocyanins on glucose homeostasis. These impacts were observed through many potential mechanisms in organs such as intestine, liver, muscle, adipocytes and pancreatic β-cells, along with their effects on gut microbiota [214,215]. Studies on animal models of diabetes have shown that anthocyanins can improve the diabetic phenotype by a range of mechanisms including preventing pancreatic β-cell inflammation, modifying enzymes in glucose metabolism and changing adipocyte function [216]. Anthocyanins decrease hyperglycaemia and insulin resistance by mechanisms including inhibition of carbohydrate metabolising enzymes such as α-amylase and α-glucosidase, increased glucose-stimulated insulin secretion by pancreatic β-cells and regulation of liver function to improve insulin resistance and changes in the gut microbiota [217]. Anthocyanins may also decrease hyperuricaemia, which is tightly linked to hyperglycaemia [217]. In insulin-resistant HepG2 cells, mulberry anthocyanin extract increased glucose consumption, glucose uptake and glycogen content [218]. Mulberry anthocyanin extract at 50 and 125 mg/kg/day doses also decreased fasting blood glucose, serum insulin, leptin, triglyceride and cholesterol concentrations and increased adiponectin levels in db/db mice [218]. These effects of anthocyanins were further improved in the presence of metformin. In mice, a combination of anthocyanins (100 mg/kg/day) and metformin (50 mg/kg/day) improved blood glucose concentrations, insulin resistance and organ damage as well as increasing beneficial bacteria in the gut microbiome and short-chain fatty acid content [219].

Reversal of changes in the gut microbiota in diabetes is a possible mechanism for improved metabolic health. Obese mice fed a high-fat high-sucrose diet and blueberry anthocyanins with faecal transplantation lost body weight and improved insulin resistance [220]. Diabetic patients have a decreased abundance of short-chain-fatty-acid-producing bacteria and tryptophan-metabolite-producing bacteria and an increased abundance of branched-chain amino-acid-synthesising bacteria and sulphate-metabolising bacteria. The unbalanced gut microbiota and microbial metabolites may be altered by interventions such as faecal transplantation, metformin or acarbose treatment, or probiotic supplementation [221].

The IGF axis has been suggested as a potential target for the treatment of diabetes and insulin resistance. Treatment with anthocyanins (320 mg/day for 12 weeks) in 56 patients with untreated fasting hyperglycaemia decreased blood glucose and C-peptide concentrations and increased blood IGFBP-4 fragments, suggesting that anthocyanins can improve glucose homeostasis by activating the IGF system [222].

Intestinal absorption of anthocyanins is through both sGLT1 and GLUT2 transporters. In addition, in animal models and isolated cell studies rather than in human clinical trials, anthocyanin treatment increased GLUT4 protein levels and translocation in peripheral tissues, which may alleviate obesity- and diabetes-induced metabolic dysfunction [223]. Further, anthocyanins and protocatechuic acid modulated intestinal glucose homeostasis in mice by increasing GLP-1 secretion and decreasing ileum expression of dipeptidyl peptidase IV [224]; these findings need to be reproduced in diabetic humans.

### 4.6. Protecting the Kidneys

Chronic kidney disease is a major public health problem with an estimated global prevalence of 13.4%, including around 5 to 7 million people needing renal replacement therapy [225]. The disease has high morbidity and mortality and no cure. Kidney function decrease can be slowed by dietary and lifestyle adjustments, especially in people with hypertension or diabetes, as well as managing cardiovascular risk, reducing the risk of infection and preventing acute kidney injury [226]. Gut dysbiosis has been suggested as a cause of chronic kidney disease [116], thus specific age-related changes in the gut microbiota may amplify the development of kidney damage [227]. Changes in the gut microbiota are associated with changes in intestinal permeability and the development of a leaky gut. Nutritional interventions such as dietary fibre may be protective in the development of a leaky gut by the production of short-chain fatty acids, while dietary fats may worsen gut function by production of lipopolysaccharides [228]. The altered gut microbiota in chronic kidney disease produces increased amounts of kidney toxins such as indoxyl sulfate, p-cresyl glucuronide, p-cresyl sulfate and indole-3-acetic acid, which then undergo increased systemic translocation to produce oxidative stress injury to the kidney [229].

Many nutritional constituents, including anthocyanins, slow down the inflammatory process in chronic kidney disease to potentially delay progression of the disease [230]. A range of polyphenols, including anthocyanins, increase gut microbiota such as *Bifidobacteria* spp. and *Lactobacillus-Enterococcus* spp., leading to protection of the intestinal barrier and decreased colonic inflammation in kidney disease. In addition, the high antioxidant responses to anthocyanins should further decrease intestinal damage [124]. These results from animal studies on kidney function have not been translated to humans, although plant-dominant low-protein diets are recommended to improve patient outcomes in chronic kidney disease [116].

### 4.7. Decreasing Obesity

The increase in the prevalence of obesity over the last 50 years has been described as a pandemic that increases the risk of many diseases including cardiovascular disease, diabetes, fatty liver, dementia and osteoarthritis [231]. For anthocyanins to be effective in chronic diseases such as obesity, they must act on relevant target cells. There is good evidence that anthocyanins interact with adipocytes, endothelial cells, inflammatory cells, hepatocytes, intestinal cells and gut microbiota, but they do not act on platelets, skeletal muscle cells, hepatic stellate cells or pancreatic β-cells [232].

A systematic review with meta-analysis of randomised clinical trials concluded that anthocyanin supplementation of 300 mg/day or less for 4 weeks was sufficient to lower body mass index and body weight, with the greatest decrease in people from the Middle East [233]. An extract from black rice mainly containing cyanidin 3-glucoside given to 47 obese postmenopausal Korean women for 12 weeks decreased lower trunk fat and total body fat percentage, possibly by reducing body fat accumulation and increasing lipolysis [234].

The anti-obesity responses to anthocyanins are likely to be a combination of different mechanisms including reduction of oxidative stress, inflammation and lipogenesis to increase lipolysis and thermogenesis and regulate satiety [235] and reversing changes in the gut microbiota. Actions of ROS that lead to obesity include regulation of adipocyte differentiation, mitochondrial dysfunction, increased endoplasmic reticulum stress, decreased lipolysis and lipogenesis, inflammation, altered adipokine production and over-activation of the sympathetic nervous system. Thus, natural products including anthocyanins that scavenge ROS could act on multiple mechanisms to prevent or reverse obesity [236]. As changes in the gut microbiota may initiate and maintain obesity-associated inflammation, suppression of the microbiota changes by anthocyanins may be relevant in reducing body fat accumulation [237]. Further, gut metabolites such as short-chain fatty acids from dietary sources may prevent obesity while metabolites from protein in the distal colon such as ammonia, phenols and branched-chain amino acids might worsen metabolic health [238].

The interplay between inflammation and obesity and its regulation by anthocyanins suggests that natural products containing anthocyanins are a strategy to reduce obesity-related chronic conditions [7]. As an example, the anthocyanins in Queen Garnet plums at a dose of approximately 200 mg/day for 4 weeks decreased body weight by 0.6 kg in healthy individuals together with increased blood adiponectin and decreased blood leptin concentrations [239].

### 4.8. Increasing Bone Repair

Continual bone remodelling requires formation of new bone by osteoblasts and removal of old bone by osteoclasts. Chronic debilitating disorders of bone function and repair include osteoarthritis [240], most notable for degradation of the articular cartilage and synovial membrane inflammation causing pain and loss of function, and osteoporosis including decreased bone density and quality increasing the risk of fracture [241].

Anthocyanin-containing fruits have shown promise in reducing the symptoms of arthritis in animal models and in human cells in culture [242]. In mice made osteoarthritic by destabilisation of the medial meniscus, cyanidin (50 mg/kg/day for 8 weeks) was protective by regulating the Sirt6/NF-κB signalling axis [243]. Further, cyanidin suppressed interleukin-1β-induced inflammatory changes in human chondrocytes. Anthocyanins from purple corn showed anti-inflammatory effects on AGE-induced human articular chondrocytes by inactivation of the NF-κB and MAPK signalling pathways [244]. Davidson’s plum containing cyanidin 3-glucoside (8 mg/kg/day) reduced obesity-induced degeneration of knee cartilage in rats with diet-induced metabolic syndrome [67]. In osteoporosis, bone regeneration may be stimulated by anthocyanins by stimulating bone formation and inhibiting bone resorption [245]; examples include peonidin 3-glucoside and cyanidin [246]. Anthocyanins may also alter bone remodelling in osteoporosis by epigenetic regulation of osteoblast differentiation and apoptosis, and bone mineralisation [247]. Further, purple corn anthocyanins and protocatechuic acid produced anti-inflammatory effects on advanced glycation end-products in human articular chondrocytes by inactivation of the NF-κb and MAPK signalling pathways [244].

The hypothesis of a “gut–joint axis” has been proposed to connect the changes in the gut microbiota and osteoarthritis factors such as age, gender, metabolism, central nervous system and joint injury [246,248]. Decreasing serum lipopolysaccharides and inflammatory responses by chronic ampicillin and neomycin treatment of osteoarthritic mice changed the microbiota and improved the signs of osteoarthritis [249]. The gut microbiota may alter bone metabolism and absorption and so changes in the microbiota may be a potential intervention to improve osteoporosis [250].

Treatment options for osteoarthritis and osteoporosis may then include altering bone formation and removal, decreasing inflammation and reversing gut microbiota changes with anthocyanins. Further studies are needed to determine whether increased dietary intake of anthocyanins in humans is associated with a decreased risk of developing osteoarthritis or osteoporosis.

### 4.9. Protecting and Repairing the Gastrointestinal Tract

Inflammatory bowel disease (IBD) as a chronic relapsing-remitting gastrointestinal disease has been used as a case study of the evolution of modern diseases by a description of the changing epidemiological patterns from the Industrial Revolution and projected to 2050 [251]. The gut microbiota in IBD patients shows decreases in beneficial bacteria and increases in pathogenic bacteria including changes that may precipitate relapse [252]. Restoring dysbiosis by increasing the production of short-chain fatty acids from dietary fibre by the microbiota may be useful to support therapy of IBD [253]. Dietary anthocyanins may alter the bacterial metabolism within the intestines and so reduce inflammation, for example in ulcerative colitis [254]. In addition, anthocyanins such as cyanidin 3-glucoside and their phenolic metabolites may improve the structure and function of the intestinal barrier and reduce oxidative stress to reduce IBD [255].

*Helicobacter pylori* is a major cause of human gastric disease around the world, especially gastric ulcers. Berry extracts containing anthocyanins produced antimicrobial activity against *H. pylori* in a high-throughput bacterial assay [256]. Black rice extract containing cyanidin 3-glucoside inhibited the biogenesis of virulence proteins in *H. pylori* and decreased apoptosis of *H. pylori*-infected cells [257]. Further, reduction of oxidative stress and inflammation may play a role in the reduction of peptic ulcers caused by ethanol and non-steroidal anti-inflammatory drugs and increased healing of acetic-acid-induced ulcers by malvidin [258]. In DSS-induced IBD in rats, cyanidin 3-glucoside and extracts of Queen Garnet plums at 8 mg/kg/day effectively reversed gastrointestinal symptoms [259] to a similar extent as sulphasalazine (~350 mg/kg/day) [260].

### 4.10. Moderating Physiological Changes in Exercise

Increased oxidative stress, inflammation, muscle damage, fatigue and fat oxidation may decrease exercise performance, for example in cycling [261]. Fruit-derived anthocyanins at doses of 8–3600 mg/day for up to 8 weeks reduced these responses to exercise and increased NO production to improve vascular function and muscle oxygenation. In addition, functional and subjective recovery after exercise was improved by anthocyanins probably due to their antioxidant and anti-inflammatory effects [262].

### 4.11. Protection against Cancer

Anthocyanins can exert effects in colorectal and breast cancer in animal studies whereas these results have not been replicated in human studies [263,264]. Anthocyanins can help in preventing DNA damage from oxidative stress in the initial stage of tumour formation. Other effects of anthocyanins may be through the inhibition of proliferation of cancer cells and migration of metastatic cells [265]. Anthocyanins interfered with cell signalling pathways related to cell growth and differentiation, apoptosis, oxidative stress and inflammatory responses in cell culture studies [266,267]. Three important signalling pathways have been identified in these chemopreventive effects of anthocyanins—AMP-activated protein kinase, PI3K/AKT/mTOR and JAK-STAT pathways [263]. Lack of suitable responses in human studies in preventing or reversing various cancers may suggest the need for further human studies in cancer patients with appropriate doses and duration of treatment.

### 4.12. Moderating Ageing

Ageing is associated with changes in physiological systems. Older adults are prone to developing chronic diseases, including cardiovascular disease, cancer, neurodegenerative disorders and osteoporosis [123]. As mentioned in the previous sections, anthocyanins can help in preventing these complications. Thus, it can be hypothesised that anthocyanins will be helpful in preventing these ageing-associated changes. Vision and eye health are also impacted during the ageing process and anthocyanins have shown beneficial effects on glaucoma when administered at 50 mg/day for up to 2 years [268]. Anthocyanins from pomegranate have shown anti-ageing effects on the skin with improved skin permeation in aged humans [269].

## 5. Further Roles for Anthocyanins in Society

The traditional Mediterranean diet characterised by increased plant foods such as fruits and vegetables has been widely studied for over 60 years for its health benefits [270]. More recently, the term “superfruit” has entered the popular vocabulary as a marketing strategy to identify exotic fruits with underutilised health benefits including açai, acerola, camu-camu, goji berry, jaboticaba, jambolão, maqui, noni and pitanga [271]. While these fruits contain a wide range of phytochemicals, it is no surprise that all contain anthocyanins as well as flavonoids and proanthocyanidins. This section will suggest that a wider usage of fruits, vegetables, cereals and agri-waste as sources of anthocyanins could sustainably improve health throughout the world. These sources of anthocyanins have been used to treat a wide range of diseases both in traditional and recent clinical practice. Some of these sources are widely available while others are very localised, suggesting a wider potential use of these locally utilised sources of anthocyanins. As increased inflammation and oxidative stress as well as changes to the gut microbiota are inter-related and commonly reported in chronic human diseases, it would be expected that interventions which act on these mechanisms, as described for anthocyanins in the previous section, will be useful to prevent or reverse these chronic diseases.

Strawberries and blueberries as common sources of dietary anthocyanins are widely consumed around the world. Beneficial effects on inflammation, cardiovascular disease, metabolic parameters, cognitive function and mental health have been summarised from 16 human intervention studies [169]. Additional potential therapeutic benefits of blueberries, based mainly on animal or cell studies, have included actions on lung function, bone strength, vision and degenerative diseases [272]. Other less well-known fruits and cereals with potential for prevention or treatment of chronic diseases that contain similar amounts of anthocyanins include black chokeberries [273], Saskatoon berries [274], jaboticaba berries [61], black rice [275], and coloured wheat [276] as well as a wide range of tropical minor fruits [277]. These anthocyanins could be extracted using novel techniques. As an example, using natural deep eutectic solvents is a green method for extraction of phytochemicals. Blueberries, when extracted with a natural deep eutectic solvent, produced a ready-to-use extract which presented higher oral bioavailability for anthocyanins in rats compared to organic solvent extract [278].

Anthocyanins can serve as natural colourants due to their intense colour. This provides an alternate source of colour in the food industry [279]. Synthetic colourants, being associated with hyperkinesis, allergic pathologies and neurological disorders in children, can easily be replaced with anthocyanins, thus providing food colour and functional properties [279]. Smart food packaging can detect and record the changes during the life cycle of packaged foods or their surroundings, which is useful to indicate the safety of food or the probable complications during storage and transport [280]. One such application of anthocyanins in the smart packaging area is the combination of sago starch and anthocyanins from red cabbage (*Brassica oleracea*), which is high in anthocyanins. As the colour of anthocyanins changes according to the pH of the environment, the colour of the smart film can be used to detect the pH changes of food or other material [281].

Purple sweet potato contains acylated anthocyanins which are more stable than other non-acylated anthocyanins, leading to pH resistance, heat resistance, lower photosensitivity and overall stability [87]. Nanoparticle encapsulation of anthocyanins using chitosan showed improved in vivo antioxidant properties indicating the potential of developing suitable delivery systems [282].

## 6. Limitations in the Current Studies

Based on the health benefits described above, anthocyanins can play crucial roles in improving health in many chronic diseases. Thus, increased commercial production of stable anthocyanins is an important research topic to be addressed. Using fruits, cereals, vegetables and agri-wastes for isolating these stable anthocyanins can provide added value to the growers of these crops. Currently, these strategies have not been achieved and need further improvements in the extraction knowledge of these valuable compounds. Using agri-waste and processing wastes from juicing or wine industry waste would provide a cost-effective method which can help in reducing the cost of the final product.

Further improvements in human studies of anthocyanins are needed. Dose–response relationships are required to be established for anthocyanins in a range of chronic diseases so that effective doses can be established. These effective doses can then be tested in large cohort trials to establish health benefits of anthocyanins, which may help nutraceutical and pharmaceutical industries to develop over-the-counter medications that can help in reducing the burden of chronic diseases in the society. Further, correlation needs to be established between the study methodologies, animals or human participant differences, doses used and study duration. Studies of fruits, vegetables or cereals containing anthocyanins should establish the profiles of anthocyanins for matching the doses of these compounds, which will help establish effective doses of anthocyanins to be used in various studies.

## 7. Conclusions

Anthocyanins are present in a wide range of fruits, vegetables and cereals, many of which are underutilised as interventions to improve health. Further, recovery of anthocyanins from agri-waste is rarely practised. Anthocyanins mediate a range of pharmacological effects, most likely due to changing the gut microbiota, decreasing oxidative stress, inflammation and neuroinflammation, and increasing the neuropeptides IGF-1 and cGP. The rapidly increasing literature on pharmacological responses to anthocyanins indicates that these compounds protect and maintain the structures and functions of the organs in the body. Based on the health benefits highlighted in this review, anthocyanins have great potential for the development of pharmaceutical and nutraceutical products. Continuing studies are therefore justified in tapping new sources of anthocyanins and clarifying the therapeutic benefits of these compounds.
